# Pulse-Type Influence on the Micro-EDM Milling Machinability of Si_3_N_4_–TiN Workpieces

**DOI:** 10.3390/mi11100932

**Published:** 2020-10-13

**Authors:** Valeria Marrocco, Francesco Modica, Vincenzo Bellantone, Valentina Medri, Irene Fassi

**Affiliations:** 1 STIIMA CNR, Institute of Intelligent Industrial Technologies and Systems for Advanced Manufacturing, National Research Council, Via P. Lembo 38/F, 70124 Bari, Italy; francesco.modica@stiima.cnr.it (F.M.); vincenzo.bellantone@stiima.cnr.it (V.B.); 2 ISTEC CNR, Institute of Science and Technologies for Ceramics, National Research Council, Via Granarolo, 64-48018 Faenza (RA), Italy; valentina.medri@istec.cnr.it; 3 STIIMA CNR, Institute of Intelligent Industrial Technologies and Systems for Advanced Manufacturing, National Research Council, Via A. Corti 12, 20133 Milan, Italy; irene.fassi@stiima.cnr.it

**Keywords:** ceramic composite, micro-EDM milling, pulse discrimination

## Abstract

In this paper, the effect of the micro-electro discharge machining (EDM) milling machinability of Si_3_N_4_–TiN workpieces was investigated. The material removal rate (MRR) and tool wear rate (TWR) were analyzed in relation to discharge pulse types in order to evaluate how the different pulse shapes impact on such micro-EDM performance indicators. Voltage and current pulse waveforms were acquired during micro-EDM trials, scheduled according to a Design of Experiment (DOE); then, a pulse discrimination algorithm was used to post-process the data off-line and discriminate the pulse types as short, arc, delayed, or normal. The analysis showed that, for the considered process parameter combinations, MRR was sensitive only to normal pulses, while the other pulse types had no remarkable effect on it. On the contrary, TWR was affected by normal pulses, but the occurrence of arcs and delayed pulses induced unexpected improvements in tool wear. Those results suggest that micro-EDM manufacturing of Si_3_N_4_–TiN workpiece is relevantly different from the micro-EDM process performed on metal workpieces such as steel. Additionally, the inspection of the Si_3_N_4_–TiN micro-EDM surface, performed by SEM and EDS analyses, showed the presence of re-solidified droplets and micro-cracks, which modified the chemical composition and the consequent surface quality of the machined micro-features.

## 1. Introduction

Silicon nitride-based ceramics constitute a class of structural materials characterized by high strength, fracture toughness, thermal shock resistance, wear resistance, low coefficient of friction, and hardness. These ceramics can often be sintered by adding electrically conductive reinforcements, such WC, MoSi_2_, TiN, TiC, TiCN, TiB_2_ and ZrN, with variable content, thus obtaining electro-conductive ceramic composites [1,2,3]. This feature allows for the manufacture of these materials via non-contact technologies such as electrical discharge machining (EDM). In particular, EDM has shown feasibility for the realization of free form features and shapes required in high-temperature and aggressive environments for the production of various heating elements (e.g., ceramic glow plugs, igniters, ceramic heaters [4]. Moreover, due to the fact of their biocompatibility, some of these ceramic composites were investigated for the realization of load-bearing prostheses and bone mini-fixation devices [3,4,5,6]. In this field, micro-EDM displayed a high potential for the fabrication of micro-free-form features in Si_3_N_4_–TiN ceramic composite workpieces needed to realize such medical components [7].

Issues related to micro-EDM manufacturing of Si_3_N_4_–TiN ceramic composite have been already explored by the research community. Indeed, machining performance and surface quality were analyzed by Liu et al. [8] in relation to different machining regimes and to the manufacturing of a mesoscopic gas turbine. From the process viewpoint, the main results demonstrated that when a relaxation-type pulse generator was chosen for the micro-EDM process, faster machining and optimized material removal rate (MRR) and low tool wear rate (TWR) could be accomplished at the expense of surface quality and roughness. In particular, surface quality inspection showed that chemical reactions, triggered by the spark occurrence, induced a modification of the composite structure and also took part in the material removal mechanism. Subsequently, Liu et al. [9] presented an analysis on the micro-EDM capability of manufacturing Si_3_N_4_–TiN ceramic composites, considering different machining regimes. In that work, the authors presented evidence of the variability ascribed to the pulse shape and duration of the discharge waveforms, not entirely due to the process setting. Therefore, also in this case, Si_3_N_4_–TiN material seemed to have played an active role in the material removal process, almost independently of the machining regimes considered for the analyses. It was evident that the optimization of micro-EDM related to ceramic composites deserved further investigation, since the relatively low value of the electrical conductivity of such material, the chemical composition of the matrix, and the consequent intrinsic inhomogeneity of the composite introduced several challenges. Very recently, a work by Selvarajan et al. [10] pointed out the effect of micro-EDM process parameters (voltage, current, pulse on time, and pulse off time) on MRR and surface quality related to two Si_3_N_4_-based composites. The results showed once more the challenge of assessing optimized process parameters and accomplishing the required feature accuracy.

In the last decade, several authors have proposed alternative approaches to investigate micro-EDM process more thoroughly. In particular, the monitoring of voltage (gap and/or open) and discharge current waveforms became established to improve the general understanding of the micro-EDM process. However, the majority of the papers dealing with the micro-EDM monitoring approach considered metals as workpieces, and their main goal was the assessment of tool wear. For instance, the authors in Reference [11] exploited discharge monitoring during micro-EDM milling to measure tool wear per discharge and material removal per discharge. A method based on pulse counting was proposed in Reference [12] to estimate the total energy associated to discharge pulses and investigate material removal and tool wear characteristic for different micro-EDM machining types (shape-up and flat-head) and conditions (spindle rotation and tool electrode vibration). The discrimination of positive and negative parts of voltage and current waveforms was proposed in Reference [13] with the goal of estimating tool wear and tool wear error. However, the reported results held validity for relatively stable micro-manufacturing and features having small depths.

A different use of micro-EDM monitoring was proposed in Reference [14]. In this work, a first classification of pulses in relation to micro-EDM milling and wire processes was presented, and four main pulse categories were identified: normal, effective arc, transient short circuit, and complex pulses. A pulse-type discrimination strategy was also implemented and applied to micro-EDM milling of hardened steel in order to investigate the influence of process parameters on discharge shapes and process performance [15]; the study emphasized the importance of sparking gap and feed rate for the process stability and identified that tool wear increase could be associated to the increase of arc number. Recently, numerical simulations and experiments were used to evaluate the occurrence of pulse types in dependence of debris accumulation [16,17]. The studies were carried out considering reverse micro-EDM (RMEDM) and confirmed that the increase of debris quantity induced a modification of pulse shapes. In particular, the authors found different pulse orders, where the first one was identified as a normal pulse, and the other two pulse types (second and higher orders, characterized by lower voltage values) occurred more frequently as the concentration of debris increased within the sparking gap. In order to evaluate the differences among pulse types and shapes, a different approach based on the analysis of power spectral density (PSD) applied to the micro-EDM milling process was proposed considering two different workpiece materials: hardened steel [18] and Si_3_N_4_–TiN [19]. The results underlined that especially for Si_3_N_4_–TiN, the number of discharges per acquisition windows underwent a huge variability, and the discharge probability was very low compared to steel workpiece manufacturing. Moreover, different energetic contributions to the material removal process from normal pulses was observed for the micro-EDM milling of Si3N4–TiN workpieces independent of the energy settings.

Although relevant investigations of the manufacturing of Si_3_N_4_–TiN ceramic composites are currently available in the literature, the influence of discharge pulse shapes on micro-EDM performance indicators has not been fully assessed. Therefore, in the present paper, micro-EDM milling of Si_3_N_4_–TiN ceramic composite was analyzed by means of pulse-type characterization and MRR and TWR evaluation. The experiments were performed by implementing a design of experiment (DOE): the finishing regime was applied, thus implying the use of relaxation-type generator producing short pulses. The voltage and current waveforms were acquired during 54 trials resulting from the DOE, where process parameters, frequency (F) and gap and pulse width (W), were varied. The pulse classification was obtained by an off-line pulse discrimination strategy. The post-processed data related to pulse types and distribution were then put in relation to MRR and TWR. Finally, chemical and surface characterization of the Si_3_N_4_–TiN ceramic composite workpiece, before and after the micro-EDM process, is also reported and discussed.

## 2. Material, Micro-EDM Settings, and Design of Experiment (DOE)

**Figure 1:**
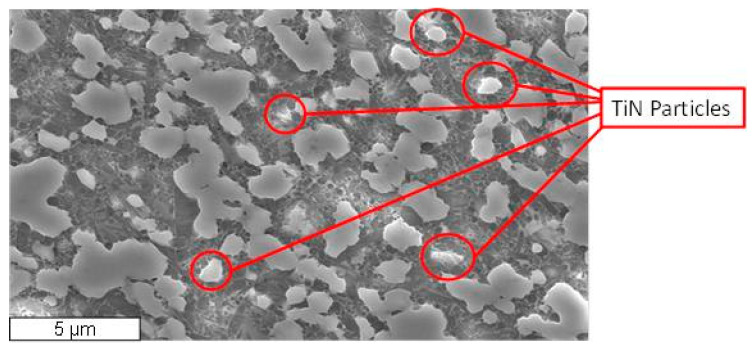
SEM micrograph of a mirror polished and plasma-etched surface of the Si_3_N_4_-35 vol% composite: dark grains are Si_3_N_4_ particles, while coarser, light grey grains are TiN particles.

The workpiece considered in this study was a sintered billet (25 mm in diameter) of Si_3_N_4_–TiN composed by a mixture of commercial raw powders. A TiN (grade C, HC Starck Ltd., Munich, Germany) 35% in volume was used as secondary electro-conductive phase and Y_2_O_3_ (grade C, HC Starck Ltd., Munich, Germany) and Al_2_O_3_ (Ultra-High Purity, Baikowski Chemie SA, Poisy, France) were used as sintering aids. The electrical resistivity was equal to 5.88 × 10^−4^ Ω cm. Figure 1 reports the SEM micrograph of the mirror polished and plasma-etched surface of the Si_3_N_4_–TiN-35 vol% composite: the typical microstructure of silicon nitride, composed of elongated silicon nitride grains in a matrix of nearly equiaxed sub-micrometric grains, was highlighted.

As highlighted in Figure 1, TiN particles are coarser (from 1 to 5 μm) than the silicon nitride grains. The grain boundary phase, consisting of silicates and oxynitrides of the cations from sintering aids, locates preferentially at triple points, and a continuous film of amorphous phase is present at the interfaces between Si_3_N_4_ grains. Furthermore, after cutting the sintered billet by diamond tool machining (DTM), two different Ra were measured: a longitudinal surface roughness equal to Ra = 0.16 µm and a transversal surface roughness equal to Ra = 0.30 µm.

The basic feature used to implement the experimental plan was a micro-channel 5 mm long, 50 µm deep, and 0.42 mm wide, manufactured onto the sintered Si_3_N_4_–TiN workpiece. The micro-EDM machine was the Sarix SX 200. The tool was a tungsten carbide (WC) cylindrical rod having a nominal diameter of 0.4 mm; hydrocarbon oil was used as dielectric fluid in the experiments. All technological parameters adopted for the trials are reported in Table 1.

For Sarix SX 200, E is an index identifying the pulse generator type selected for the machining. In this case, E110 indicates finishing regime actuated by RC relaxation-type generator capable of producing short pulses. The OCV is the open voltage value, i.e., the maximum voltage achievable before discharge. Since the micro-EDM milling approach is implemented via a layer-by-layer strategy, a layer thickness (LT) of 1 μm was set. Frequency (F) indicates the frequency of the micro-EDM pulse generator, while pulse width (W) indicates the time interval in which the micro-EDM generator is disconnected from the tool and the workpiece and the discharge occurs within the sparking gap. Finally, Gap is the index related to the servo control loop responsible for the sparking gap stability (i.e., the distance between the tool and the workpiece). In order to estimate the statistical impact of the parameters’ variability on machining performance and pulse distribution, a general full factorial Design of Experiment (DOE) was implemented: the varied process parameters were frequency (F) and pulse width (W) and Gap, which were grouped in 18 sets of parameters (SoP); three replicas of the experimental plan were performed, resulting in a total of 54 trials.

## 3. Micro-EDM Monitoring Setup and Pulse Discrimination Strategy

**Figure 2:**
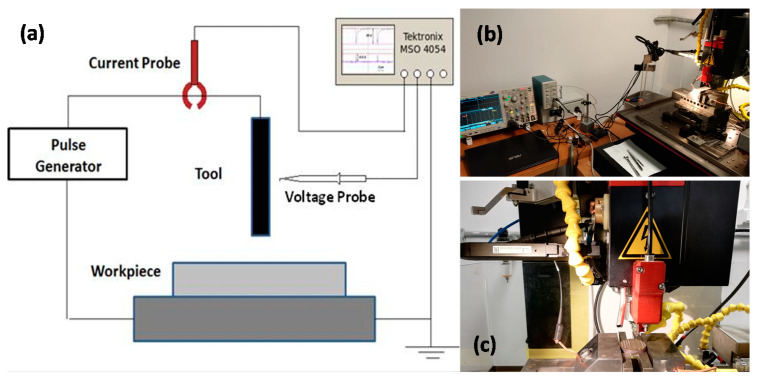
(**a**) Sketch of the micro-EDM monitoring setup; (**b**) actual monitoring setup connected to the Sarix SX 200 via voltage and current probes; (**c**) voltage and current probes mounted on the Sarix SX 200.

The sketch of the micro-EDM monitoring setup, along with the actual machining setup, is reported in Figure 2.

The acquisition of the voltage and current waveforms was done by means of two probes—a standard voltage probe and a Tektronix TCP312 current probe with a bandwidth of 100 MHz. The voltage probe was placed close to the tool tip in order to record voltage values (OCV, gap voltage). As the current probe works through Hall effect, it was hooked to the cable supplying the tool. In order to acquire and record the waveforms, both probes were connected to the oscilloscope—Tektronix MSO4054. The pulse generator was not connected to the oscilloscope.

The oscilloscope sampling frequency (Fs = 50 MHz) and the number of samples for each waveform (Ns = 10^6^) were set considering the bandwidth of signals to be acquired and the duration of the phenomenon. With this setting, the duration of each acquisition window was equal to T_acq_ = 20 ms. The acquired data were delivered to a PC through the oscilloscope USB port: due to the USB port speed, an elapsed time of 0.8 s between two subsequent windows in each trial must be considered. The acquired waveforms were then supplied as input to the off-line classification algorithm which provided the number of each pulse type as an output. The pulse types were gathered in four classes: short, arc, delayed, and normal [14,15,18,19]. The discharge events occur within the sparking gap during the time interval equal to T–W, being T = 1/F, but only if the spark condition is fulfilled. When the discharge happen, a drop of OCV to its minimum value is observed. In this case, a normal pulse is detected. If the spark condition is not favorable, OCV keeps its constant value, as no discharge occurs. Nonetheless, local machining conditions, such as the presence of unremoved debris in the sparking gap, can promote discharge events independently of the pulse generator; in this case, two types of pulses can be observed: arcs and delayed. The distinction between these two categories can be made based on the maximum voltage value. Generally, arcs have lower Vmax than delayed. Short pulses happen when both tool and workpiece electrodes come into contact due to the instantaneous local debris concentration, and they are characterized by low voltage values approaching the ground level. Short pulses do not provide any material removal process and generally produce a waste of time.

**Figure 3:**
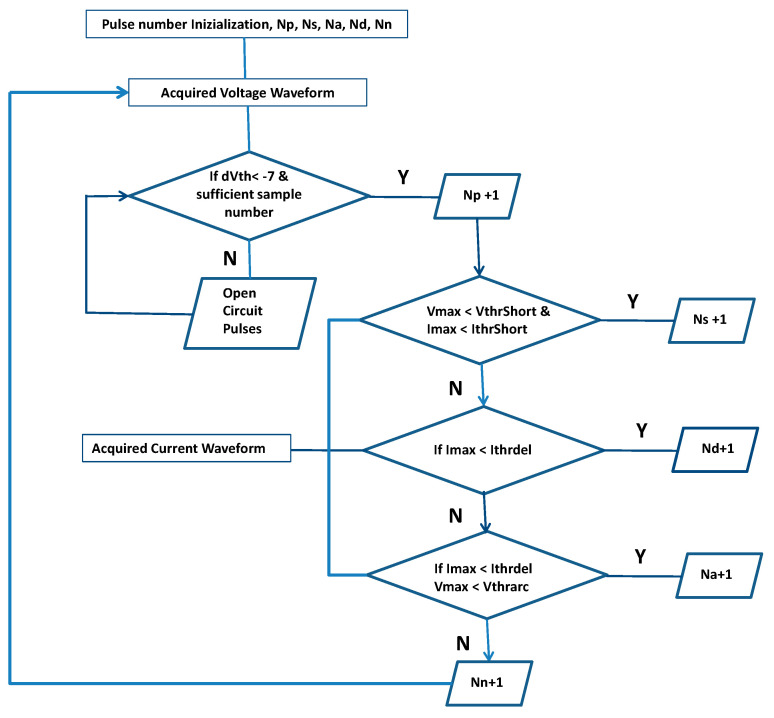
Flow chart of the discrimination strategy algorithm applied to all acquisition windows at each trial.

The flow chart of the pulse discrimination strategy is reported in Figure 3; all routines were implemented in MATLAB^®^. The beginning of the algorithm foresees an initialization phase, where all pulse type counters—N_p_ (total), N_s_ (shorts), N_a_ (arcs), N_d_ (delayed), N_n_ (normal)—are reset. Subsequently, the acquisition windows of one trial are analyzed one by one via the algorithm, to assess the presence and type of pulses. The first iteration comprises the computation of the time derivative of the V waveform (dV); then, this value is compared to a set threshold (dV_th_). If dV < dV_th_, one pulse is identified, and the total counter N_p_ is incremented by one (N_p_ + 1). If this condition is not satisfied, the program starts searching again for a pulse. If a pulse is found, the algorithm proceeds to its identification, starting from shorts: in this case, a comparison among mean values of V and I and defined thresholds V_thrShort_ and I_thrShort_ is performed. If this check succeeds, the counter N_s_ is incremented by one (N_s_ + 1). If this check fails, then the algorithm searches for delayed pulse. A delayed pulse is identified by comparing the maximum I recorded during the discharge with the defined threshold I_thrDel_. If this check succeeds the counter N_d_ is incremented by one (N_d_ + 1), otherwise the algorithm searches for arcs. The criterion for this comparison is the same as before, along with the comparison of the mean V with a programmable threshold V_thrArc_. If this check succeeds the counter N_a_ is incremented by one (N_a_ + 1), otherwise the pulse is classified as normal and the counter N_n_ is incremented by one (N_n_ + 1). At the end of the classification, the index of the derivative of the voltage array is incremented of a programmable amount (about a half of the pulse width) in order to speed up the next pulse search, and the loop starts over again to analyze the subsequent acquired observation window.

The defined current and voltage threshold values shown in Table 2.

## 4. Results

In this section, pulse-type distribution, statistical analysis, and the results regarding the influence of pulse-type number and occurrence on MRR and TWR are presented and discussed.

### 4.1. Pulse-Type Distribution

**Figure 4:**
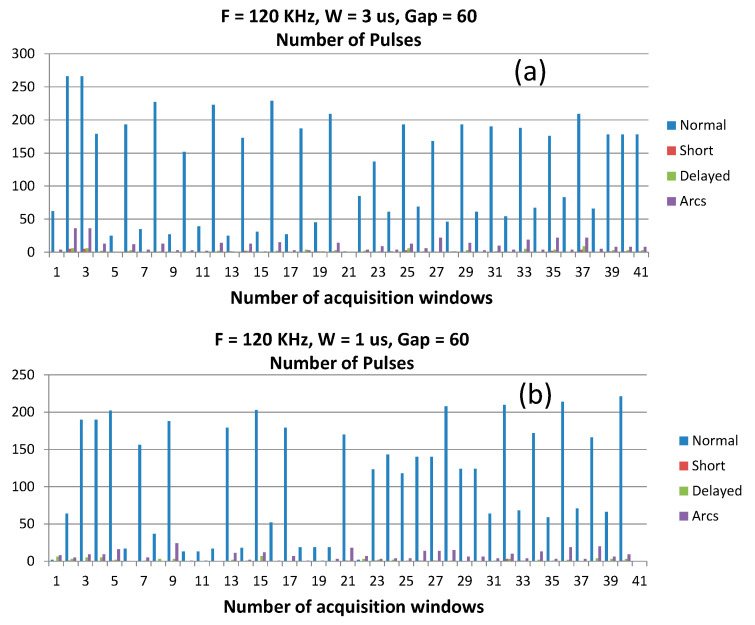
Number of normal pulses counted in the latest 40 acquisition windows for two different trials: (**a**) F = 120 kHz, W = 3 μs, G = 60; (**b**) F = 120 kHz, W = 1 μs, G = 60.

The monitoring of each trial required a certain number of acquisition windows, approximately 250 for each process. The monitored trials related to all micro-EDM processes displayed relevant variability of discharge occurrence. In order to explain this fact, we reported the count of all pulse types recorded in the last 40 acquisition windows (i.e., the last part of the machining) and pertaining two distinct trials: F = 120 kHz, G = 60, W = 3 µs and F = 120 kHz, G = 60, W = 1 µs. As shown in Figure 4, the total number of all discharges occurring in each acquisition window was very variable. This fact confirms issues about the repeatability of the micro-EDM process on this kind of ceramic composite, mainly ascribable to the workpiece itself rather than to the parameter settings. Nonetheless, it is evident that in all acquired subsequent widows, the number of normal pulses always exceeds the number of arcs, delayed, and shorts.

**Figure 5:**
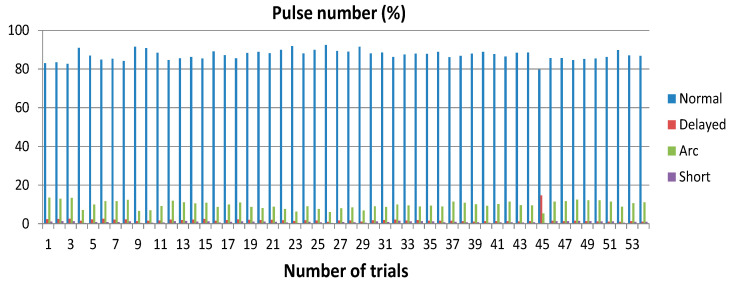
Number of normal, arc, short, and delayed pulses reported in percentage.

Figure 5 shows the average distribution in percentage of all pulse types for all trials calculated during the last part of the micro-EDM manufacturing. The histograms highlight that normal pulses are the most numerous, indicating the general stability experienced during all micro-EDM trials. This fact is also confirmed by the negligible number of shorts, arcs, and delayed pulses. It is worth stressing that an anomalous behavior was found in trial 45, which was repeated at the end of all trials.

### 4.2. Analysis of Variance: Pulse Type and Process Parameters

Before proceeding with the statistical analysis of pulse types, the analysis of variance (ANOVA) was carried out to assess the relation between the selected process parameters (F, W and Gap) and the performance indicators, MRR, and TWR. The coefficients of determination *R*^2^, which defines how much variation in the response is explained by the model, were 86.7% for MRR and 37.4% for TWR. These values suggested that the used model described quite properly the relation among F, W and Gap, and MRR, but it was not accurate in relation to TWR. It is worth stressing that the tool wear lengths measured by control touch procedure during micro-EDM milling process were generally very low (ranging between 3.5–6 µm) and this could have prevented the model from describing the statistical relation properly.

The analysis of variance was applied to the pulse types in order to identify the statistical relation with the process parameters. The coefficients of determination *R*^2^ for normal, arc, delayed, and short pulses were 81.5%, 85.7%, 85.2%, and 68.8%, respectively. In the following analysis, short pulses were not considered; since their number was low, they were not statistically relevant, as indicated by the lowest coefficient of determination, and they did not take part as a material removal mechanism.

**Figure 6:**
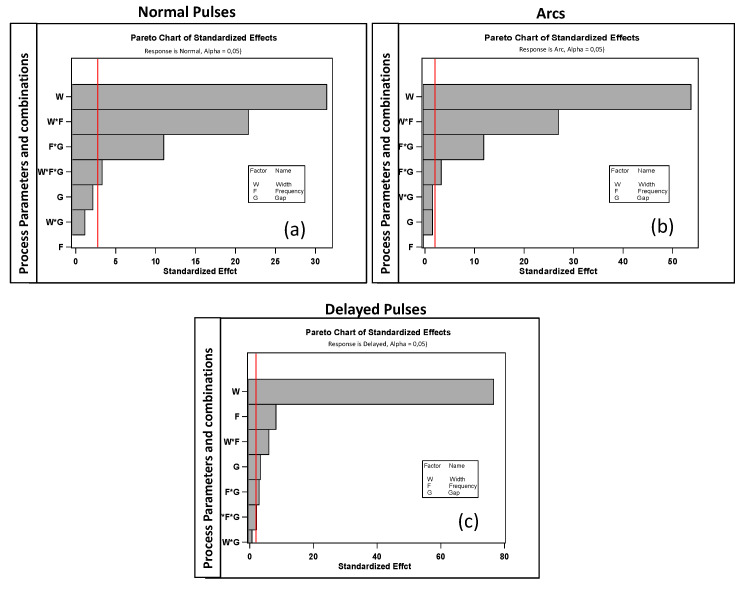
Pareto charts for (**a**) normal, (**b**) arcs, and (**c**) delayed pulses.

The first step of the analysis comprised the examination of residuals for normal, arc, and delayed pulses to assess the validity of the ANOVA method. The Pareto charts are reported in Figure 6. For all cases, the Pareto showed normal distribution and homogeneity of variances, thus confirming the validity of the assumptions for the ANOVA method application.

As evident from the charts, all pulses were influenced by combinations of the chosen process parameters. Only delayed pulses displayed less sensitivity to gap, while it was mainly influenced by W and F. All regression equations obtained from the statistical analysis and related to normal, arc, and delayed pulses are reported in Appendix A.

**Figure 7:**
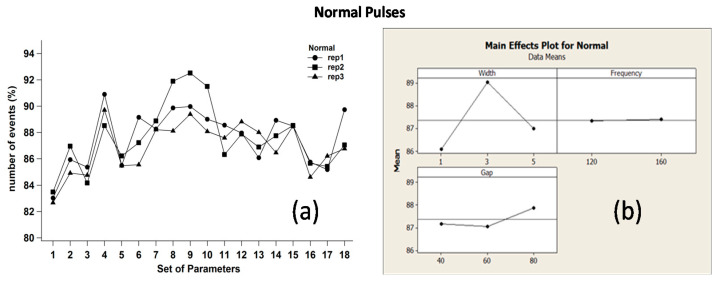
(**a**) Replica diagrams and (**b**) main effects plot related to normal pulses.

**Figure 8:**
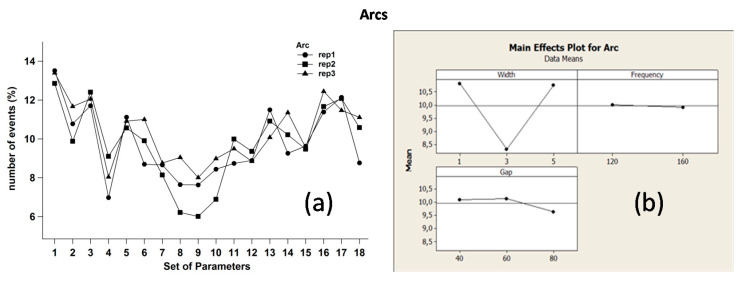
(**a**) Replica diagrams and (**b**) main effects plot related to arc pulses.

**Figure 9:**
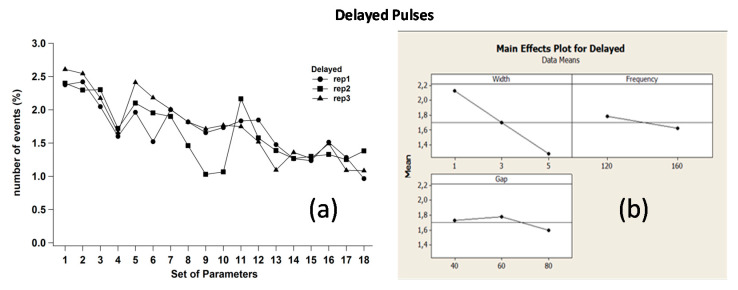
(**a**) Replica diagrams and (**b**) main effects plot related to delayed pulses.

Replica diagrams and main effects plots were computed and reported in Figure 7, Figure 8 and Figure 9. Figure 7a,b refer to normal pulses; the graphs shows that their number increased when W was equal to 3 μs. In this case, the generator was completely recharged within W, so that the rest of the period t–W was reserved for discharge events. As one can notice, in the considered range of values, parameter F had almost no effects on pulse occurrence, whereas higher gap values (farther distances between tool and workpiece) favored normal pulses.

Figure 8a,b refer to arcs, where the minimum number of such a pulse type was detected in correspondence of W = 3 μs and Gap = 80, thus confirming that these parameter values could actually provide a stable process. Nonetheless, also in this case, F seemed to have negligible impact on arcs. It is worth noting that normal and arc pulses exhibited a dual response in relation to the process parameter effects, i.e., an increase in normal pulses should correspond a decrease in arcs.

The plots related to delayed pulses are reported in Figure 9a,b.

Delayed pulses have different dependences on W, F and Gap compared to the previous pulse types. First, they exhibit a strong linear dependence on W. Moreover, if F is reduced, the discharge probability time t = T–W increases slightly, thus promoting the probability for a delayed to happen. Furthermore, for a higher gap value, the delayed pulse occurrence is certainly reduced. In other words, when the tool and the workpiece are farther from each other, the required energy to allow spark ignition is the highest possible, i.e., set OCV. However, when maximum voltage is applied to the load, it is more likely to trigger normal pulses rather than delayed, which are characterized by lower OCV values. The result is that delayed number decreases. Furthermore, the plots show quite clearly that the delayed–Gap relation is similar to the arcs.

### 4.3. Relation between Pulse Types and Machining Performance

**Figure 10:**
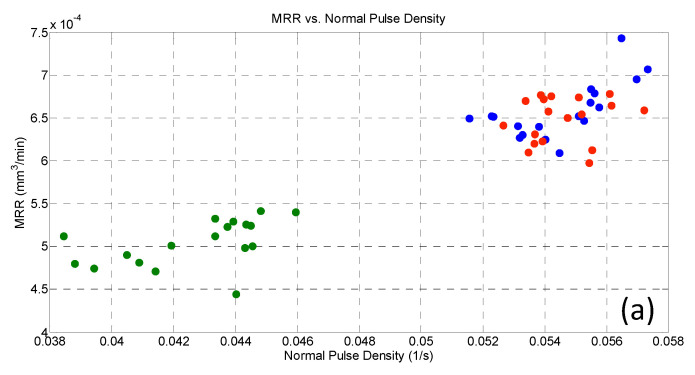

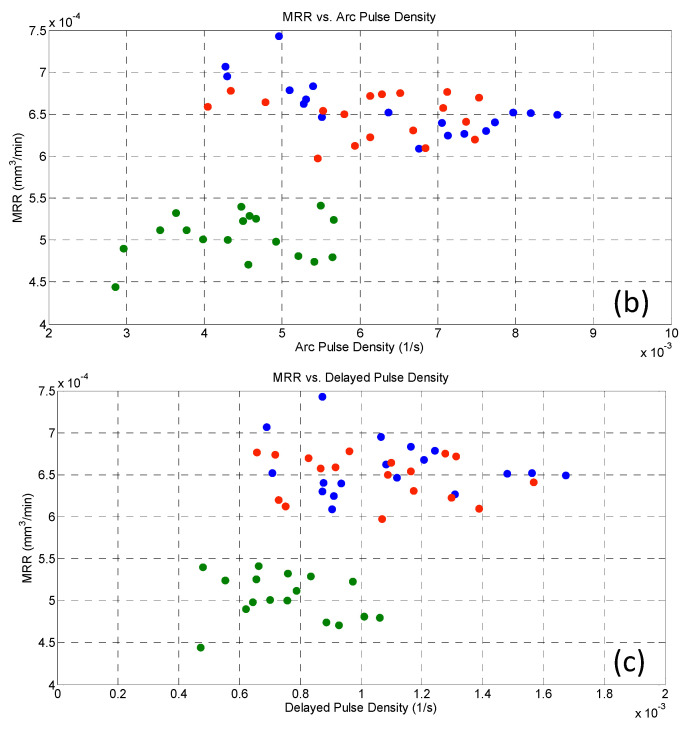
MRR versus (**a**) normal, (**b**) arc, and (**c**) delayed pulse density: blue dots refer to gap = 40, red dots to gap = 60, and green dots to gap = 80.

**Figure 11:**
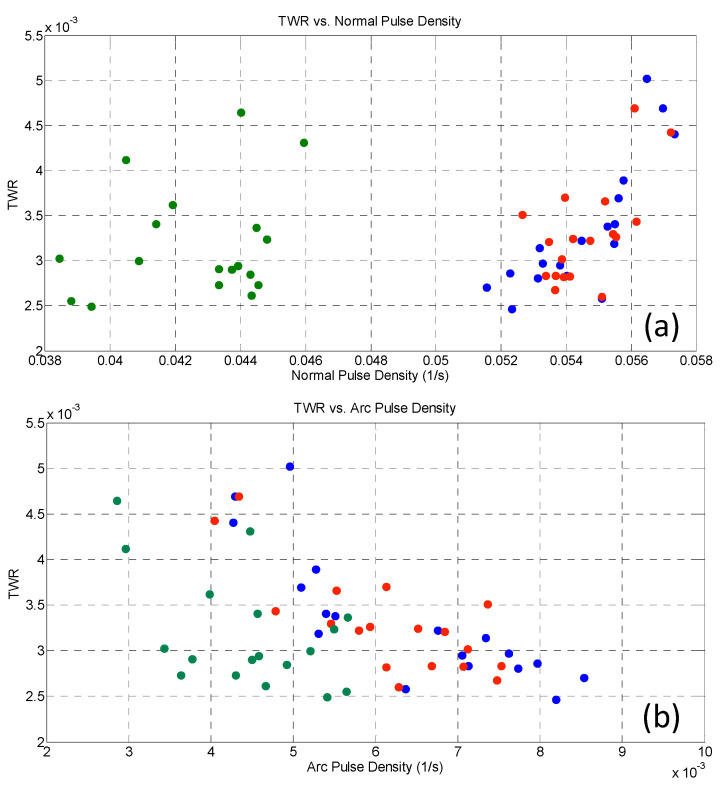

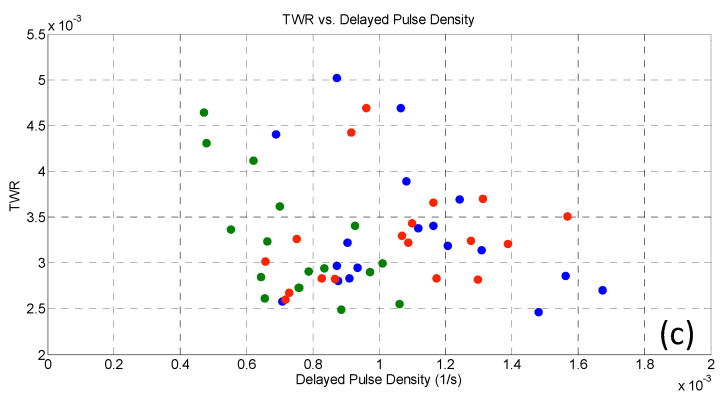
TWR versus (**a**) normal, (**b**) arc, and (**c**) delayed pulse density: blue dots refer to gap = 40, red dots to gap = 60, and green dots to gap = 80.

In previous works [15,18], the monitoring, pulse discrimination, and statistical analysis were performed on hardened steel workpieces with similar process parameter settings. In particular, those previous results suggested that a higher number of normal pulses had a positive effect on process stability and machining speed, so that MRR results would be good, while TWR increased its value, thus following a dual trend with respect to MRR. It was also found that as arcs increased in number and occurrence, MRR had a beneficial turn, and TWR worsened. Considering this roadmap, similar results were partly expected in the present analysis, as the role of the ceramic workpiece composition would certainly affect the final results. In order to represent MRR and TWR as a function of pulse type number and occurrence, a normalized pulse density (NPD) was defined according to the following equation:

NPD = average (N_PulseType_/T_acq_) (1)

where N_PulseType_ is the number of each pulse type (normal, arc, short or delayed) recorded in the last 25 µm of micro-channel manufacturing, and T_acq_ is the duration of the acquisition window. Figure 10 and Figure 11 depict the corresponding average values of MRR and TWR. The diversity in the dots color indicates the process parameter chosen to discriminate the behavior, in this case, Gap.

Figure 10a shows that MRR increases as the occurrence of normal pulses increases as well. When Gap is higher, a lower number of overall pulses is expected as the tool and workpiece are farther from each other. Moreover, in this condition, the erosion process was slower thus decreasing MRR. On the contrary, arcs (Figure 10b) did not induce any variability on MRR, while a slight decrease of it was observed when a delayed occurrence was higher (Figure 10c). Reasonably, by considering the nature of delayed pulses, the increase in the machining times could explain this trend. Nonetheless, these results reveal that arcs and delayed did not affect MRR significantly; so, even though these pulse types are capable of removing material, their occurrence was neither problematic nor positive to the process, as long as their percentage was kept smaller than normal pulses.

Figure 11 reports TWR behavior in the function of normal, arc, and delayed normalized pulse density.

First, it must be pointed out that the overall recorded TWR values are very small, in particular if these results are compared to those obtained with hardened steel (TWR is one order of magnitude higher for steel, and it ranged between 0.02–0.04 [15,18]). However, the relation between TWR and normal pulses confirms the expected trend, i.e., higher pulse occurrence leads to higher tool consumption. Conversely, a peculiar trend can be observed in Figure 11b: when arcs increase, TWR decrease, and this is in contrast with the expected results. Indeed, arcs are capable of removing material from both workpieces and tools, and an increase of TWR should have actually been measured. It was also verified that the tool wear volume was actually lower. Two reasons may explain these results: the first relies on the energy density of arcs, which was lower than normal pulses, and so less material could have been removed from both electrodes. Furthermore, a protective layer, generated by the melting, decomposition, and evaporation of Si_3_N_4_ particles during the micro-EDM process, was deposited on the tool surface and prevented the tool from more severe wearing. Finally, Figure 11c displays the trend for delayed pulses which is akin to that reported for arcs, thus suggesting a similarity of the nature for such pulses.

### 4.4. Surface Evaluation of Si_3_N_4_–TiN Workpiece before and after the Micro-EDM Process

**Figure 12:**
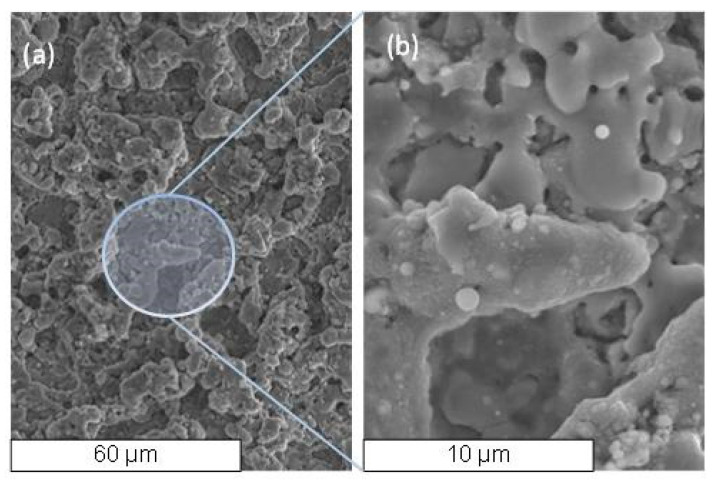
SEM micrograph of the EDM surface (**a**) and magnified (**b**).

The SEM and EDS analyses were also performed to evaluate the surface quality after micro-EDM milling of the Si_3_N_4_–TiN ceramic composite. Figure 12 evidences a foamy and porous structure, as also observed in Reference [8] with many spark-induced craters and melt-formation droplets appearing on the composite surface after micro-EDM.

The super-positioning of craters with varying diameters, which is not clearly distinguishable, derives from the material removal mechanism, linked to the interaction between the materials characteristics and chemical reactions triggered by the erosion process. The quantity of material that solidified and adhered to the surface is a function of the composition and microstructure of the starting ceramic materials, whereas the re-solidified droplets are almost entirely due to the TiN particles; conversely, Si_3_N_4_ particles are removed by evaporation. We also observed that, for a length of about 1 mm, the tool tip was covered by a protective layer, typically composed of Si_3_N_4_ particles. The presence of micro-cracks was due to the thermal expansion mismatch between silicon nitride, titanium nitride (3.0·10-6 °C-1 and 9.4·10-6 °C-1, respectively) and re-solidified particles induced by the plasma breakdown. It can be also noticed that, since the material exhibited a degree of porosity, this could have affected the discharge probability, thus reducing the number of total discharges. For sake of completeness, surface roughness Ra of the machined micro-channels was also measured after micro-EDM processes: the Ra values ranged between 0.77–0.98 μm.

**Figure 13:**
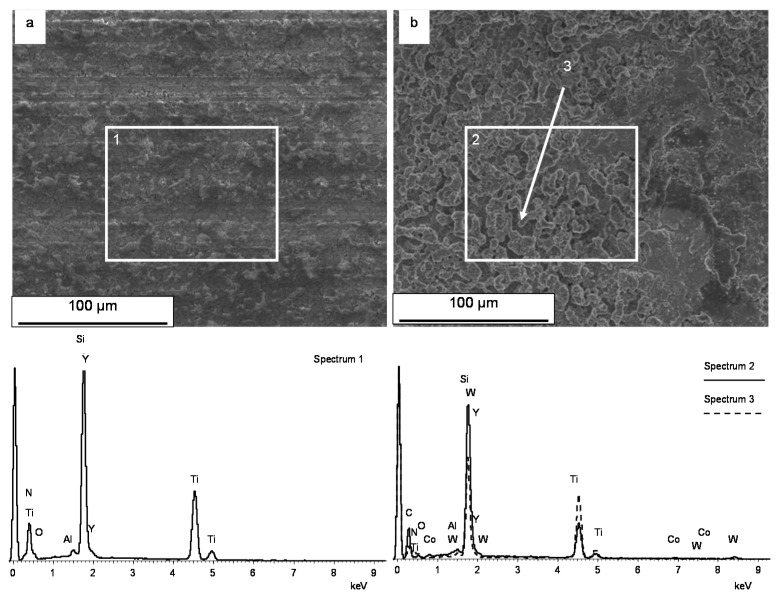
SEM-EDS characterization of Diamond Tool Machining (DTM) (**a**) and micro-EDM (**b**) surfaces.

Elemental composition was checked by EDS and reported in Figure 13, where DTM and micro-EDM surfaces are compared. The element concentration is explicated in Table 3.

From the analysis of the micro-EDM surfaces, the presence of C and W belonging to the tool was detected. Moreover, a marked decrease of N due to the fact of its evaporation during the erosion process was evident with respect to the DTM surface (Table 3). The elemental composition was checked on the micro-EDM surfaces also by point EDS analysis on a merging particle. Besides the presence of C and W, Co was also detected due to the contribution of the tool (Co is commonly used in WC composition), while N was absent. It can be noticed that the Si/Ti atomic ratio was not constant, as it depends on the dimension of the checked area and on the surface modification due to the preferential ablation/oxidation of Si_3_N_4_ or TiN during the micro-EDM process.

## 5. Discussion

The experimental results highlighted that the micro-EDM machinability of Si_3_N_4_–TiN ceramic composite workpieces differ drastically from experienced practice on metal workpieces. In particular, the statistical analysis of the pulse types in relation to process parameters showed that the triggering of normal, arc, and delayed pulses cannot be simply controlled through the setting of a single process parameter, but rather through a combination of them, such as pulse width W, frequency F and Gap. Moreover, when the pulse type occurrence is put in relation to micro-EDM performance indicators, it is clear that the MRR results were reasonably influenced by normal pulses, while arcs and delayed had a negligible effect on it. On the contrary, TWR exhibited very low values and did not display the common response expected from the different pulse types; in particular, arcs and delayed pulses did not affect TWR negatively.

## 6. Conclusions

The micro-EDM machinability of Si_3_N_4_–TiN ceramic composite workpieces was investigated by putting in relation pulse-type distribution with process parameters and performance indicators. To this aim, a Design of Experiment was implemented by considering the variation of pulse width W, frequency F and Gap. During the experiments, voltage and discharge current waveforms were acquired: the data were then post-processed to discern pulse type (i.e., normal, arcs, delayed, and shorts) via the pulse discrimination strategy. First, the pulse distribution showed that normal pulses were the most numerous, whereas other pulse types occurred very sporadically. The collected data were then statistically evaluated to elicit the relation existing among process parameter combinations and pulse-type distribution. The statistical analysis proved statistical reliability only for normal, arc, and delayed pulses, whereas short pulses were neglected in the discussion due to the numerical and statistical irrelevance. Pareto charts, regression equations, replica diagrams, and main effects plots revealed that all pulses were influenced by combinations of all selected process parameters.

The final analysis of the influence between pulse type and process performance indicators (MRR and TWR) showed that:

- MRR values increase as the number of normal pulses grows. Arcs and delayed pulses do not produce any positive or negative effects on MRR. A reasonable explanation is found in their low number and in their effective contribution to the material removal process.
- TWR displays very low values in all trials. Despite this, it was observed that the increase in normal pulses leads to the increase in TWR, as expected, whereas the increase in arcs and delayed pulses induces a peculiar decrease in TWR.

Finally, it was shown that the Si_3_N_4_–TiN workpiece features intrinsic degrees of electrical-conductivity inhomogeneity before machining as assessed by SEM and EDS evaluation. Additionally, same analyses performed on the micro-EDM surface displayed re-solidified droplets and micro-cracks, induced by chemical mechanism, which witnessed a surface roughness increase with respect to un-machined surface. Indeed, the surface roughness Ra measured after micro-EDM ranges between 0.77–0.98 μm, whereas the starting Ra of the workpiece ranges between 0.16 and 0.30 μm.

## Figures and Tables

**Table 1:** The micro-electro discharge machining (EDM) milling process parameter settings.

Micro-EDM Process Parameter	Unit	Values
Energy (E)	Index	110 (finishing, RC generator)
Open Circuit Voltage (OCV)	V	100
Layer Thickness (LT)	µm	1
Frequency (F)	kHz	120–160
Pulse Width (W)	µs	1–3–5
Gap	Index	40–60–80

**Table 2:** I and V threshold settings.

Threshold Value	(% of OCV and Imax)
I_thr_	50% Imax (A)
I_thr_short_	10% Imax (A)
V_thr_short_	30% OCV (V)
V_thr_arc_	95% OCV (V)

**Table 3:** EDS element concentration.

Element	Spectrum 1: DTM Window (Atomic %)	Spectrum 2: EDM Window (Atomic %)	Spectrum 3: EDM Point (Atomic %)
C K	-	35.21	66.68
N K	45.01	10.91	-
O K	9.36	10.50	3.23
Al K	0.77	0.59	0.59
Si K	26.34	18.42	16.22
Ti K	17.79	23.41	9.45
Co K	-	-	0.51
Y L	0.75	0.46	0.60
W M	-	0.50	2.71

## Appendix A

Since two of the considered factors (W and Gap) had three levels, a general full factorial design was planned for the experiments. The corresponding regression equations obtained by the statistical analyses for normal, arcs, and delayed pulses are reported below:

W = Width, G = Gap, F = Frequency

Normal = 87.379 − 1292 W_1 + 1667 W_3 − 0.375 W_5 − 0.042 F_120 + 0.042 F_160 − 0.198 G_40 − 0.305 G_60 + 0.503 G_80 − 1.455 W × F_1 120 + 1.455 W × F_1 160 + 0.677 W × F_3 120 − 0.677 W × F_3 160 + 0.779 W × F_5 120 − 0.779 W × F_5 160 + 0.495 W × G_1 40 + 0.055 W × G_1 60 − 0.550 W × G_1 80 + 0.141 W × G_3 40 − 0.015 W × G_3 60 − 0.126 W × G_3 80 − 0.636 W × G_5 40 − 0.039 W × G_5 60 + 0.676 W × G_5 80 − 0.968 F × G_120 40 + 0.840 F × G_120 60 + 0.128 F × G_120 80 + 0.968 F × G_160 40 − 0.840 F × G_160 60 − 0.128 F × G_160 80 − 0.858 W × F × G_1 120 40 + 0.761 W × F × G_1 120 60 + 0.097 W × F × G_1 120 80 + 0.858 W × F × G_1 160 40 − 0.761 W × F × G_1 160 60 − 0.097 W × F × G_1 160 80 − 0.203 W × F × G_3 120 40 − 0.238 W × F × G_3 120 60 + 0.441 W × F × G_3 120 80 + 0.203 W × F × G_3 160 40 + 0.238 W × F × G_3 160 60 − 0.441 W × F × G_3 160 80 + 1.061 W × F × G_5 120 40 − 0.523 W × F × G_5 120 60 − 0.538 W × F × G_5 120 80 − 1.061 W × F × G_5 160 40 + 0.523 W × F × G_5 160 60 + 0.538 W × F × G_5 160 80 (A1)

Arc = 9.963 + 0.848 W_1 − 1.643 W_3 + 0.795 W_5 + 0.051 F_120 − 0.051 F_160 + 0.136 G_40 + 0.181 G_60 − 0.318 G_80 + 1.169 W × F_1 120 − 1.169 W × F_1 160 − 0.580 W × F_3 120 + 0.580 W × F_3 160 − 0.589 W × F_5 120 + 0.589 W × F_5 160 − 0.298 W × G_1 40 − 0.170 W × G_1 60 + 0.468 W × G_1 80 − 0.141 W × G_3 40 + 0.022 W × G_3 60 + 0.120 W × G_3 80 + 0.439 W × G_5 40 + 0.148 W × G_5 60 − 0.587 W × G_5 80 + 0.721 F × G_120 40 − 0.632 F × G_120 60 − 0.089 F × G_120 80 − 0.721 F × G_160 40 + 0.632 F × G_160 60 + 0.089 F × G_160 80 + 0.668 W × F × G_1 120 40 − 0.632 W × F × G_1 120 60 − 0.036 W × F × G_1 120 80 − 0.668 W × F × G_1 160 40 + 0.632 W × F × G_1 160 60 + 0.036 W × F × G_1 160 80 + 0.016 W × F × G_3 120 40 + 0.274 W × F × G_3 120 60 − 0.290 W × F × G_3 120 80 − 0.016 W × F × G_3 160 40 − 0.274 W × F × G_3 160 60 + 0.290 W × F × G_3 160 80 − 0.684 W × F × G_5 120 40 + 0.358 W × F × G_5 120 60 + 0.326 W × F × G_5 120 80 + 0.684 W × F × G_5 160 40 − 0.358 W × F × G_5 160 60 − 0.326 W × F × G_5 160 80 (A2)

Delayed = 1.7033 + 0.4235 W_1 − 0.0001 W_3 − 0.4234 W_5 + 0.0827 F_120 − 0.0827 F_160 + 0.0260 G_40 + 0.0799 G_60 − 0.1059 G_80 + 0.1428 W × F_1 120 − 0.1428 W × F_1 160 − 0.0747 W × F_3 120 + 0.0747 W × F_3 160 − 0.0681 W × F_5 120 + 0.0681 W × F_5 160 − 0.0924 W × G_1 40 + 0.0834 W × G_1 60 + 0.0090 W × G_1 80 + 0.0165 W × G_3 40 + 0.0237 W × G_3 60 − 0.0403 W × G_3 80 + 0.0759 W × G_5 40 − 0.1071 W × G_5 60 + 0.0312 W × G_5 80 + 0.1045 F × G_120 40 − 0.0603 F × G_120 60 − 0.0441 F × G_120 80 − 0.1045 F × G_160 40 + 0.0603 F × G_160 60 + 0.0441 F × G_160 80 + 0.0708 W × F × G_1 120 40 − 0.0340 W × F × G_1 120 60 − 0.0368 W × F × G_1 120 80 − 0.0708 W × F × G_1 160 40 + 0.0340 W × F × G_1 160 60 + 0.0368 W × F × G_1 160 80 + 0.1106 W × F × G_3 120 40 − 0.0560 W × F × G_3 120 60 − 0.0545 W × F × G_3 120 80 − 0.1106 W × F × G_3 160 40 + 0.0560 W × F × G_3 160 60 + 0.0545 W × F × G_3 160 80 − 0.1814 W × F × G_5 120 40 + 0.0900 W × F × G_5 120 60 + 0.0913 W × F × G_5 120 80 + 0.1814 W × F × G_5 160 40 − 0.0900 W × F × G_5 160 60 − 0.0913 W × F × G_5 160 80. (A3)
